# Scaling and maintenance of corneal thickness during aging

**DOI:** 10.1371/journal.pone.0185694

**Published:** 2017-10-06

**Authors:** Takenori Inomata, Alireza Mashaghi, Jiaxu Hong, Takeshi Nakao, Reza Dana

**Affiliations:** 1 Schepens Eye Research Institute, Massachusetts Eye and Ear Infirmary, Department of Ophthalmology, Harvard Medical School, Boston, MA, United States of America; 2 Juntendo University Faculty of Medicine, Department of Ophthalmology, Tokyo, Japan; 3 Leiden Academic Centre for Drug Research, Faculty of Mathematics and Natural Sciences, Leiden University, Leiden, The Netherlands; Cedars-Sinai Medical Center, UNITED STATES

## Abstract

Corneal thickness is tightly regulated by its boundary endothelial and epithelial layers. The regulated set-point of corneal thickness likely shows inter-individual variations, changes by age, and response to stress. Using anterior segment-optical coherence tomography, we measure murine central corneal thickness and report on body size scaling of murine central corneal thickness during aging. For aged-matched mice, we find that corneal thickness depends on sex and strain. To shed mechanistic insights into these anatomical changes, we measure epithelial layer integrity and endothelial cell density during the life span of the mice using corneal fluorescein staining and *in vivo* confocal microscopy, respectively and compare their trends with that of the corneal thickness. Cornea thickness increases initially (1 month: 114.7 ± 3.0 μm, 6 months: 126.3 ± 1.6 μm), reaches a maximum (9 months: 129.3 ± 4.4 μm) and then reduces (12 months: 127 ± 2.9 μm, 13 months: 119.5 ± 7.6 μm, 14 months: 110.6 ± 10.6 μm), while the body size (weight) increases with age. We find that endothelial cell density reduces from 2 months old to 8 months old as the mice age and epithelial layer accumulates damages within this time frame. Finally, we compare murine corneal thickness with those of several other mammals including humans and show that corneal thickness has an allometric scaling with body size. Our results have relevance for organ size regulation, translational pharmacology, and veterinary medicine.

## Introduction

Size is a critical property of biological systems and is tightly regulated [1]. Body size determines the metabolic rate of organisms [2, 3], interactions of organisms with their environment [4, 5] and is related to biological diversity and population size [6]. How does body size relate to the size of internal organs, what determines size of internal organs, and how internal organs respond to environmental stresses are fundamental questions in biology [7–9].

Allometry, a term coined by Julian Huxley and Georges Tessier in 1936, applies to the phenomenon of relative growth. Organs may have higher growth rate than the whole body (positive allometry), identical growth rate with the whole body (isometry) or lower relative growth rate (negative allometry) [10]. It is noteworthy that studies on allometry are not limited to analyzing age-related changes, the so-called ontogenetic allometry, but also include analysis of inter-individual and inter-species size variations, termed as static and evolutionary allometry respectively.

The eye has been subject to allometric analysis. Axial length of vertebrate eyes obeys a logarithmic relationship with body weight with a negative allometric scaling [11]. Visual organs in human grow to its 80% of adult size by age 4 [12]. Early in life, the orbit size changes with age and doubles its birth weight by 7–8 years of age when it reaches the adult size [13]. The size of an emmetropic human adult eye does not depend on sex or age [14]. Whether eye components also follow size rules similar to the whole eye remains to be studied.

The cornea forms the anterior segment of the eye and is the eye’s primary light-focusing structure. Here, we ask how central cornea thickness changes during development and aging in laboratory mouse and how it scales with body size. We determine how the scaling is affected by sex, and how it depends on species. Finally, we perform a systematic literature study and compare body size scaling of murine corneal thickness to several other mammals including humans.

## Materials and methods

### Mice, husbandry and anesthesia

1–14 month old C57BL/6 (H-2b) and BALB/c (H-2d) female and male mice were purchased from Charles River Laboratories (Wilmington, MA, USA). Mice were housed in a specific pathogen-free environment at the Schepens Eye Research Institute animal facility. They were aged in our AAALAC-certified vivarium in a standard 12:12- hour light–dark cycle and fed irradiated diet (Teklad global 19% protein extruded Rodent Diet 2918, Harlan Laboratories, Indianapolis, IN, USA). Mice were weighed by weight scale.

Anesthesia was administered intraperitoneally by ketamine/xylazine solution at a dose of 120 mg/kg body weight and 20 mg/kg body weight, respectively. Under these conditions, the eyes of mice are naturally wide open and in a stable position, with pupils pointing laterally and upward.

All animals were treated according to the guidelines established by the Association for Research in Vision and Ophthalmology (ARVO) Statement for the Use of Animals in Ophthalmic and Vision Research and Public Health Review, and all procedures were approved by the Institutional Animal Care and Use Committee of the Schepens Eye Research Institute.

### Corneal thickness measurement

**Fig 1:**
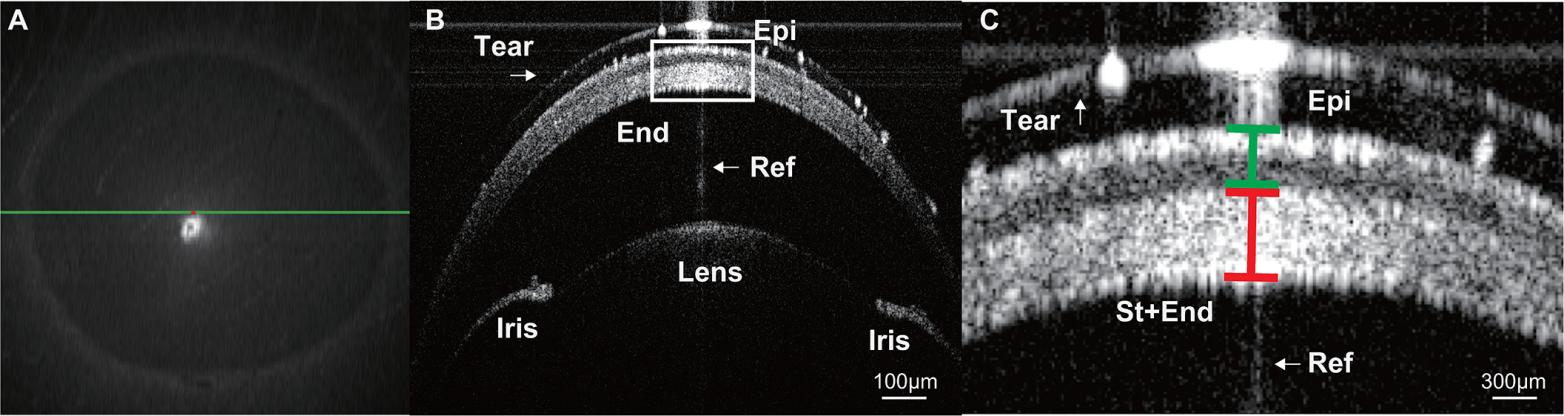
Corneal thickness measurement by AS-OCT. (A) En face projection view (green line shows the position of B-Scan). (B and C) Cross-sectional image of the cornea visualizing different corneal layers (B-Scan). Higher magnification is shown (C). Tear: Tear layer, Epi; Corneal epithetium, Sto: Corneal stroma, End: Corneal endothelium. Ref: corneal vertex reflection.

Images of the anterior segment were taken by anterior segment-optical coherence tomography (AS-OCT; Bioptigen, Durham, NC, USA) in order to determine the corneal thickness. For high resolution central corneal cross-sectional scans (scan range; 3.0mm, scan resolution; 1000, 100 length) were obtained by the radial scan mode at each time point. We aligned the position of cornea by the real-time display used for guidance (Fig 1A). The position of the cornea was adjusted until the intensity peaks corresponding to the cornea were detected and maximized. The center of the scan pattern was aligned with the corneal vertex reflection [15] visualized on the OCT images (Fig 1B). Corneal epithelial thickness (Epi) and, the total amount of corneal stroma (St) and corneal endothelial thickness (End), were measured by the supplied software (Fig 1C).

### Corneal endothelial cell density measurement

*In vivo* confocal microscopy (IVCM), the Heidelberg Retina Tomograph (HRT) / Rostock Cornea Module (Heidelberg Engineering GmbH, Heidelberg, Germany) was used to examine endothelial cell density (ECD) in the cornea. Mice were anesthetized and placed on the microscope stand and the eyes were coated with Genteal gel (Novartis, St. Louis, MO, USA). Images were taken covering an area of 400×400 μm^2^ and axial optical resolution of 1 μm/pixel. Then, ECD areas were analyzed quantitatively using ImageJ.

### Corneal fluorescein staining

Corneal fluorescein staining (CFS) and the National Eye Institute grading system (Bethesda, MD) were used to evaluate corneal epithelial damage caused by DED [16]. Briefly, 1 ml of 2.5% fluorescein (Sigma-Aldrich) was applied into the lateral conjunctival sac of the mice and after 3 minutes corneas were examined with a slit lamp biomicroscope under cobalt blue light. Punctate staining was recorded in a masked fashion with the standard National Eye Institute grading system of 0–3 for each of the five areas of the cornea—central, superior, inferior, nasal, and temporal.

### Allometric analysis

In allometric analysis, the relationship between the two measured quantities is typically expressed as a power law function which expresses a scale symmetry: Y = kX^α^, or in a logarithmic form: Log(Y) = αLog(X) + Log(k) [17]. Thus, we fit a linear function to the log/log plot of our data and report the slope, α, as the estimated allometric coefficient.

### Statistical analysis

**Fig 2:**
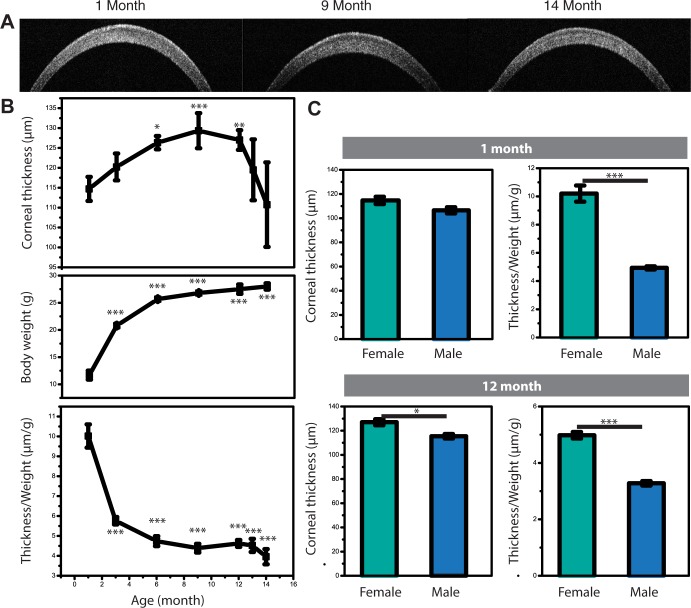
Age-related changes in corneal thickness. (A) Representative OCT images of corneas from female BALB/c mice. (B) Corneal thickness, body weight and corneal thickness/ weight versus age in female BALB/c mice. (C) Corneal thickness depends on sex. Corneal thickness is compared between male and female BALB/c mice. All data were obtained from n = 10 mice/group and representative data from three independent experiments are shown. All data were compared to baseline (1 month). *p* values are calculated using one-way ANOVA with Bonferroni post hoc test, and error bars represent SEM. (*<0.05, **<0.01, ***<0.001).

**Fig 3:**
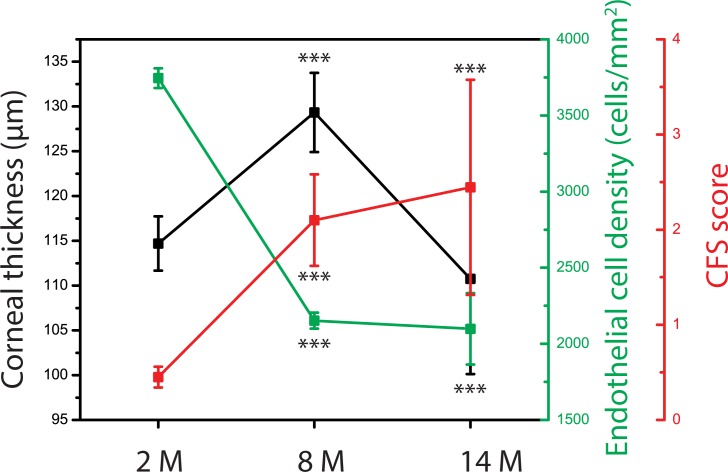
Correspondence between corneal thicknesses and the status of corneal epitheliopathy and endothelial cell layers. The data suggest that, initially, aging affects corneal thickness by changing the boundary layers that maintain the homeostasis of corneal water and electrolytes. Endothelial cell density (ECD) reduces as the mice age from 2M old to 8M old and epithelial layer accumulates damages within this time frame. We have not seen significant changes in corneal epitheliopathy and ECD (contrary to thickness data), when we assessed very old mice (14M). All data were obtained from n = 10 mice/group and representative data from three independent experiments are shown. All data were compared to baseline (2 months). We used female BALB/c mice for this analysis. *p* values are calculated using the Student’s t-test and error bars represent SEM. (***<0.001).

**Fig 4:**
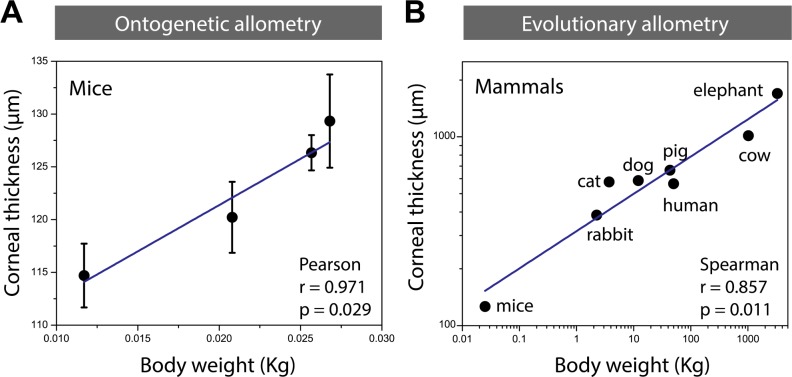
Allometric scaling for corneal thickness. (A) Allometric scaling analysis of 6-month-old female BALB/c mice. Pearson correlation coefficient was used. (B) Evolutionary allometric scaling of adult mammals (α = 0.2 ± 0.02), calculated from reported data for mice, rabbit, cat, dog, pig, human, cow and elephant. Linear regression analysis and correlation analysis were performed among body weight and corneal thickness. Spearman correlation coefficient was used. Pearson correlation analysis was used for normally distributed data and Spearman correlation analysis was adopted for the abnormally distributed data.

Significance of difference of corneal thickness, body weight and corneal thickness adjusted by weight between different groups were analyzed by one-way ANOVA with Bonferroni post hoc test (Fig 2), and corneal thickness, endothelial cell density and CFS scores were compared to baseline levels by Student’s t-test (Fig 3) using Prism software (GraphPad, San Diego, CA, US). Data are presented as mean ± standard error of mean (SEM) and considered statistically significant at *p* <0.05. Linear regression analysis and correlation analysis were performed among body weight and corneal thickness using Origin V8.5 SR1 software (OriginLab corporation, Northampton, MA, US) and the built-in statistical packages (Fig 4). Pearson correlation analysis was used for normally distributed data and Spearman correlation analysis was adopted for the abnormally distributed data.

## Results

### Cornea thickness changes by age

We measured cornea thickness for BALB/c female mice at different ages ranging from one month (114.7 ± 3.0 μm) to 14 months (Fig 2A and 2B). We identified two phases: (i) the thickness increases initially (6 months: 126.3 ± 1.6 μm), and then reaches a maximum (9 months: 129.3 ± 4.4 μm); (ii) we then observed a reduction in the corneal thickness (12 months: 127 ± 2.9 μm, 13 months: 119.5 ± 7.6 μm, 14 months: 110.6 ± 10.6 μm). The body size (weight) showed a distinct trend (Fig 2B). For young ages, we observed an increase in the body weight by age that saturates at nearly 6 months (1 month: 11.7 ± 0.6 g, 3 month: 20.8 ± 0.3 g, 6 month: 25.5 ± 0.3 g, 9 months: 27.8 ± 0.7 g, 12 month: 27.5 ± 0.9 g, 13 month: 26.5 ± 0.9 g, 14 month: 28.0 ± 0.6 g). Weight normalized thickness (Fig 2B) follows a trend comprised of an initial decline (1 month: 10.0 ± 0.6 μm/g, 3 month: 5.7 ± 0.2 μm/g, 6 month: 5.0 ± 0.1 μm/g, 9 months: 4.5 ± 0.2 μm/g), a plateau and a decline after one year (12 month: 4.5 ± 0.2 μm/g, 13 month: 4.5 ± 0.3 μm/g, 14 month: 4.0 ± 0.4 μm/g).

We then assessed the contributions of different cornea layers to the overall thickness change. We observed that stroma and endothelium contribute to the thickness change during adulthood and also late in life (S1 Fig). Finally, we assessed epithelial integrity and ECD to see whether age-related changes of corneal thickness are correlated with structural changes of the boundary epithelial and endothelial layers (Fig 3 and Panels A-C in S2 Fig). Initially, aging affects corneal thickness by changing the boundary layers that maintain the homeostasis of corneal water and electrolytes. ECD reduces as the mice age from 2 months to 8 months (2 months: 3745.3 ± 64.8 cells/mm^2^, 8 months: 2310.8 ± 46.7 cells/mm^2^) and epithelial layer accumulates damages within this time frame (2 months: 0.5 ± 0.1, 8 months: 2.1 ± 0.5, 14 months: 2.4 ± 1.1). Contrary to thickness data, we have not seen significant changes in corneal epitheliopathy and ECD (14 months: ECD; 2098.3 ± 234.5 cells/mm^2^), when we assessed very old mice (14M).

Next we asked if corneal thickness depends on sex and strain. We compared the corneal thickness of 1-month old mice and we observed no difference between the two sexes (Fig 2C). However, for 1-year-old mice we observed significantly thinner cornea in male animals. The difference between sexes became clearer when we normalized the thickness by body weight. To determine if mice of different strains differ in their corneal thickness, we studied two aged- and sex-matched strains, BALB/c and C57BL/6J mice. We observed no significant difference in corneal thickness between the two strains initially, but after normalizing the thickness with body weight, we found that C57BL/6J mice have relatively thinner corneas for their body size (S3 Fig).

### Boundary epithelial and endothelial layers change with age

To gain mechanistic insights into the dynamics of corneal thickness, we measured ECD (Panels A and B in S2 Fig). We observed that the density declines fast after one month for several weeks and then continuously decreases with a relatively lower rate for young adults until 14 months. We then normalized the density values with weight and observed that the weight-normalized thickness initially declines and then reaches a plateau at 5–6 months of age.

Next, we asked if animals with different gender differ in their ECD (Panel C in S2 Fig). We compared the ECD of 1-month old female and male mice and we observed no differences. For 1-year old mice, we observed a lower ECD for male mice as compared to the female ones. Normalizing the density values with weight, we found more dramatic difference between the genders. The male animals, both 1-month and 1-year old, had lower densities when compared to their aged-matched female counterparts.

To gain further mechanistic insights into the dynamics of corneal thickness, we measured epithelial integrity of mouse cornea and its dependency on age (Fig 3). Using CFS and slit lamp assessment, we found that the integrity of corneal epithelium is significantly impaired in 8-month old mice irrespective of gender. The corneal epithelial lining of young adult mice (2-month old) was found to be typically intact.

### Evolutionary allometry of cornea

We compared murine corneal thickness with that of other mammals with different body sizes (evolutionary allometric analysis). We extracted central cornea thickness of adult elephant, human, pig, cow, cat, dog, rabbit, and mice [18–25] as well as their reported body weights [20, 21, 23–28] from the literature, and performed allometric analysis (Fig 4). We found that the log-log plot of the corneal thickness versus body weight shows a linear trend. We extracted the allometric coefficient by fitting a linear function to the log-log plot.

## Discussion and conclusions

In this study, we measured central corneal thickness changes with age, assessed the dependence of corneal thickness on sex in young and old mice, and compared murine corneal thickness with that of other mammals. The latter provided us with a scaling relation which in turn provides us with a simple way to estimate weight/age dependency of corneal thickness in other animals by only knowing it for mice and without directly measuring it for other animals (which might be practically very difficult for certain rare species).

The changes of corneal thickness by age were still unclear in human and animals. Previous studies reported that there was no significant change in the corneal thickness over time [29, 30] and the others showed the decreased trend of corneal thickness by age [31, 32]. However, those studies did not adjust the measured thicknesses by study subjects’ body sizes (weights), which is what we performed in our study (Fig 2). The thickness has a seemingly increasing trend in younger phase [33], which follows by a decreasing trend (Fig 3), but after adjusted by weight, we clearly showed the central corneal thickness has a decreasing trend by age.

The increase in corneal thickness in old mice (8 month old) as compared to young mice (2 month old) was associated with increase in epitheliopathy and decrease in ECD (Fig 3). The changes in epithelial and endothelial function may explain, at least partially, the observed changes in corneal thickness because epithelium and endothelium maintain the hemostasis of water and materials [34] in the cornea. Due to age related changes in these layers, flux of water and materials will likely change and a new steady state and a new corresponding thickness may be reached. Our study however does not provide any functional analysis of epithelial and endothelial layer and these possible scenarios are to be tested in future studies.

To compare corneal thickness from other inbred strains of mice, we measured the central cornea thickness of both BALB/c and C57BL/6J mice (S3 Fig). Our study showed that the murine central corneal thickness was highly strain-dependent. Our data supported previous studies demonstrating that the central corneal thickness of C57BL6J mice was thinner than that of BALB/c mice under weight adjustment [25]. In addition, we revealed ECD was also strongly influenced by genetic backgrounds, suggesting that the genes may influence the physiologic attrition of ECD. Our data has great potential to increase our understanding of the ECD disorder.

This study has some limitations that should be noted. First, we only used AS-OCT for assessing corneal thickness. For accurate examination, it is preferable to evaluate corneal thickness with various machines such as AS-OCT [15] and Pentacam Scheimpflug system [35]. In clinical setting for human, previous study has reported the comparison between AS-OCT and Pentacam Scheimpflug system for assessing corneal thickness, reporting that AS-OCT and Pentacam are both reliable and reproducible for measuring corneal thickness [36], therefore we examined mice cornea thickness by AS-OCT in this study. Moreover, CFS score was used for assessing corneal epitheliopathy in this study. Although we did not evaluate the corneal structural changes by histological assessment, CFS score was used historically for the evaluation of corneal epitheliopathy for the ocular surface disease such as dry eye disease [37], which is strong correlation with aging [38–41]. Therefore, we consider CFS score was useful for assessing corneal surface epitheliopathy by aging in murine model. Finally, this study was focused on corneal thickness and not cornea size. The thickness is only a partial measure of cornea size. Allometric scaling however can be done for any length scale in our body including limb length and corneal thickness as reported in this article.

Our study has revealed dynamics of corneal thickness during the lifetime of laboratory mouse. Our study will be of interest to researchers studying aging, comparative ophthalmology and veterinary medicine. To extrapolate the results of pharmacological studies performed on mice to other animals, it is essential to understand relevant scaling relations. This is important not only for human studies but also for designing drug therapy for rare animals.

## Supporting information

S1 Fig Thickness of epithelium and, stroma and endothelium combined versus age. Female BALB/c mice were used for the OCT measurements. All data were obtained from n = 10 mice/group and representative data from three independent experiments are shown. All data were compared to baseline (1 month). *p* values are calculated using the Student’s t-test and error bars represent SEM. (*<0.05). The left panel presents the full cornea thickness, which is split into two parts, thickness of epithelium and, thickness of stroma and endothelium combined in the right panel.
(EPS)

S2 Fig Age-related changes in endothelial cell density (ECD). (A) Representative HRT images of corneal endothelial layers from female BALB/c mice. (B) Corneal ECD versus age in female BALB/c mice. (C) Dependency of ECD on sex. Corneal ECD is compared for male and female BALB/c mice. All data were obtained from n = 10 mice/group and representative data from three independent experiments are shown. All data were compared to baseline (1 month). *p* values are calculated using one-way ANOVA with Bonferroni post hoc test, and error bars represent SEM. (***<0.001).
(EPS)

S3 Fig Corneal thickness and ECD are compared for BALB/c and C57BL/6J mice. The results of OCT and HRT measurements for the two strains of mice are presented. 12-month old male animals were used for this analysis. All data were obtained from n = 10 mice/group and representative data from three independent experiments are shown. *p* values are calculated using the Student’s t-test and error bars represent SEM. (***<0.001).
(EPS)
